# The effect of syringe design and cannula dimensions on time-force curve in intravitreal injection across different drug viscosities: area under the curve and peak injection force

**DOI:** 10.1186/s40942-026-00833-2

**Published:** 2026-03-20

**Authors:** Bryan Calder Ackermann, Niklas Junker, Maximilian Friedrich, Øystein Kalsnes Jørstad, Morten C. Moe, Victor Aristide Augustin, Gerd Uwe Auffarth, Maximilian Hammer

**Affiliations:** 1 Department of Ophthalmology, University Clinic Heidelberg, Heidelberg, Germany; 2 The David J Apple® International Laboratory for Ocular Pathology, University Eye Clinic Heidelberg, Heidelberg, Germany; 3 Department of Ophthalmology, Oslo University Hospital, Oslo, Norway; 4 Faculty of Medicine, University of Oslo, Oslo, Norway; 5 Faculty of Biosciences, Heidelberg University, Heidelberg, Germany

**Keywords:** Intravitreal injection, Syringe design, Injection force, Pegcetacoplan, Bevacizumab

## Abstract

**Background:**

New intravitreal medications can offer longer treatment duration by using higher drug concentrations, which, in turn, increase viscosity. There is also a trend toward having smaller-gauge cannulas, which require a higher injection force. While the ophthalmologist cannot control viscosity, the choice of syringe may affect the force needed to administer the drug. This study compared two standard 1-ml syringes (BD Plastipak^™^ and Braun Injekt^®^-F) with a syringe featuring a reduced plunger diameter (Zero Residual^™^ 0·2 mL silicone oil-free syringe) for injecting a variety of fluids with viscosity that mimics commonly used intravitreal formulations.

**Methods:**

Injection force over time was measured for each combination of the three syringes, four fluids, and two cannulas (30 G and 33 G), and the results were compared using a two-way ANOVA, followed by Tukey’s post-hoc test. Plungers of the 1ml syringes had a diameter of approximately 4.70mm, while the smaller syringe had a plunger diameter of 2.48mm. Of the 1ml syringes, one was silicone oil lubricated (BD Plastipak™). The tested fluids were chosen to simulate bevacizumab (Balanced Salt Solution), pegcetacoplan (13.8 mg/0.1 ml of PEG-40,000), a high-viscosity solution (40 mg/0.1 ml of PEG-400), and a very high-viscosity solution (Tween 80 diluted with BSS).

**Results:**

The Zero Residual syringe required significantly lower injection forces, as quantified by both the area under the curve (AUC) and peak force. These effects became more pronounced when using smaller-diameter cannulas and increasingly viscous fluids. The BD Plastipak needed lower forces than the Braun Injekt-F.

**Conclusions:**

While silicone-oil lubrication reduced injection force, its effect was modest compared with that of plunger diameter, which was the primary determinant of the injection force required for a given viscosity and cannula diameter.

**Supplementary Information:**

The online version contains supplementary material available at 10.1186/s40942-026-00833-2.

## Introduction

Intravitreal injection (IVI) of drugs has become a widely established mode of therapy for retinal diseases such as neovascular age-related macular degeneration (nAMD), diabetic macular edema, and geographical atrophy. Unlike systemic administration, drugs do not have to cross the blood-retina barrier, resulting in higher intraocular drug concentrations [1, 2] and enhanced therapeutic efficacy.

Since their introduction, newer drug formulations have become more concentrated and viscous [3–5], while cannula diameters have been reduced to enhance patient comfort, decrease the risk of infection, and minimize drug reflux [6]. According to the Hagen-Poiseuille equation, both trends increase the force required for injection, viscosity through a linear effect and inner cannula radius through a fourth-power effect. The increased injection force potentially hinders controlled injection and can even cause the cannula to detach from the syringe [7]. The remaining variable that can be modified to control injection force lies within the syringe itself, specifically its dimensions, including plunger diameter and length. Most pharmacies use nonspecialized syringes originally designed for intravenous, subcutaneous, or intramuscular administration. While these all-purpose syringes accommodate a broad range of applications, they have not been optimized for the injection of small volumes of highly viscous substances through high gauge (narrow) cannulas. Additionally, some syringes are lubricated with a silicone oil coating to facilitate initial plunger mobilization and injection.[7]

This study aimed to quantify and compare three commonly used and available syringes, and to assess the effects of plunger diameter and silicone oil lubrication on injection performance across different cannula sizes (30 G and 33 G) and solution viscosities. The main goal of this work is to identify the most suitable syringe from currently available options in regards to injection force, when cannula size and drug viscosity are fixed.

## Methods and materials

### Syringes, cannulas and fluids

Three syringes were evaluated with four fluids of varying viscosities and two different sizes of cannulas. Each combination was measured five times, resulting in a total of 120 measurements. Of the syringes, one featured a smaller plunger diameter of 2.48 mm (Zero Residual^™^ Silicone Oil-free 0.2 ml Syringe, SJJ Solutions B.V., The Hague, Netherlands), henceforth referred to as “Syringe A”, while two had similar plunger diameters of approximately 4.70 mm, one with silicone oil lubrication (BD Plastipak^™^ 1 ml, BD Canaan, CT, USA) henceforth referred to as “Syringe B” and one without (Braun Injekt^®^-F Silicone Oil Free 1 ml Syringe, B. Braun SE, Melsungen, Germany), we refer to as “Syringe C”.

To summarize, syringes either differed by lubrication (Syringe B vs C) or by plunger diameter (Syringe A vs B/C), allowing for assessment of material-related and geometric effects.

Measurements were conducted at the David J Apple^®^ International Laboratory for Ocular Pathology, Department of Ophthalmology, University of Heidelberg, Germany. Ethical approval was not required as this study did not involve animal or human subjects.

To achieve a high number of measurements and conduct the study cost-effectively, two fluids were selected to represent the viscosities of common drug formulations and two other fluids served as high viscosity stress tests. This allowed to investigate whether results observed with lower viscosities are magnified under extreme conditions. As part of pre-study preparation, the viscosities of the fluids were determined as described in the section *Rheological measurements of fluid viscosities*. An overview of the study materials can be found in Table 1.

**Table 1:** Materials used in the study

**Syringes**	*“Syringe A”* Zero Residual Silicone Oil-free 0.2 ml Syringe (SJJ Solutions B.V., The Hague, Netherlands d = 2.48 mm	
	*“Syringe B”* Braun Injekt-F Silicone Oil Free 1 ml Syringe (B. Braun SE, Melsungen, Germany) d = 4.71 mm	
	*“Syringe C”* BD Plastipak 1 ml (BD, Canaan, CT USA) d = 4.76 mm	
**Solutions**	**Composition and Viscosity**	**Intended Clinical Equivalent**
	Balanced Salt Solution (BSS) η=27.25 mPa·s	Bevacizumab (Avastin, Roche-Genentech, San Francisco, CA, USA)
	13.8 mg/0.1 ml solution of PEG-40,000^1^ η=51.06 mPa·s	Pegcetacoplan (Syfovre®, Apellis Pharmeceuticals Inc, Waltham, MA, USA)
	40 mg/0.1 ml solution of PEG-400^2^ η=97.75 mPa·s	High viscosity solution
	1-part pure Tween-80 polysorbates to 2 parts BSS η=13656.50 mPa·s	Very-high viscosity solution
**Cannulas**	Zero Residual^=^ 30 G 13 mm (1/2”) cannula (SJJ Solutions B.V.)	
	Zero Residual^™^ 33 G 9 mm (3/8”) cannula (SJJ Solutions B.V.)	

All possible combinations of syringe, fluid and cannula were tested

d = Diameter of the syringe plunger; η = Complex Viscosity

^1^water-soluble mixture containing polyethylene glycol (PEG) with an average molecular weight of approximately 40,000 g/mol

^2^water-soluble mixture containing polyethylene glycol (PEG) with an average molecular weight of approximately 400 g/mol

For each measurement, 0.1 ml of fluid was injected. Syringes were inspected for air bubbles prior to injection to ensure consistent volume delivery across all measurements.

### Rheological measurements of fluid viscositiess

Resistance to flow was measured as fluid viscosity under standardized laboratory conditions.

Viscosity was measured using a modular compact rheometer (MCR 302e, Anton Paar Group AG, Graz, Austria) with a cone-plate measuring geometry (CP50-1, Anton Paar Group AG, Graz, Austria). After initializing the device, setting the temperature to 25 °C, and determining the zero gap, approximately 650 µl of each fluid was placed onto the measuring plate. Frequency sweep measurements were then conducted two times, with the fluid being removed and reapplied between measurements. From the resulting data, a frequency within the low-frequency plateau was selected as a representative value of the complex viscosity.

Complex viscosity at an angular velocity of 3.79 rad/s served as a representative value of viscosity and was averaged from all measurements.

An overview of the measurements is listed in Table 2.

**Table 2:** Rheological measurements of the study fluids

Solution	Intended Clinical Equivalent	Measured Complex Viscosity [mPa·s]		Mean [mPa·s]	SEM [mPa·s]
		#1	#2		
BSS	Bevacizumab	27.25	27.25	27.25	0
PEG 40,000	Pegcetacoplan	51.38	50.73	51.055	0.325
PEG 400	High viscosity solution	100.41	95.09	97.75	2.66
Tween 80 + BSS	Very-high viscosity solution	16341.00	10972.00	13656.5	2684.5

Complex viscosity displayed was measured at an angular velocity of 3.79 rad/s

SEM = Standard Error of the Mean

### Resistance force measurements

Resistance force was measured following our group’s established protocol [8], based on the method first described by Usui and Tanaka [9]. During each injection, the resistance force was measured 10 times per second using an automated digital force gauge (FV-10XY, Nidec-Shimpo Corporation, Tokyo, Japan) with the Shimpo Toriemon Force Gauge software. Afterwards, the measured force was plotted against time to determine area under the curve, representing the total resistance encountered during injection and thus, effort to expel the fluid and peak force, reflecting the maximum force a clinician could expect during injection. Additionally to representing the necessary effort for injection, AUC also allowed for better comparability across multiple measurements. By integrating the time-force curve, injections with varying force-time curves netted similar AUCs, as is illustrated in Fig. S1. In cases of a detached syringe or other issues during testing, the measurement was aborted and repeated.

The total resistance forces during the whole injection process were calculated from the area under each implantation curve in Newton-seconds [N⋅s] using numerical integration.

### Injection procedures

**Fig. 1:**
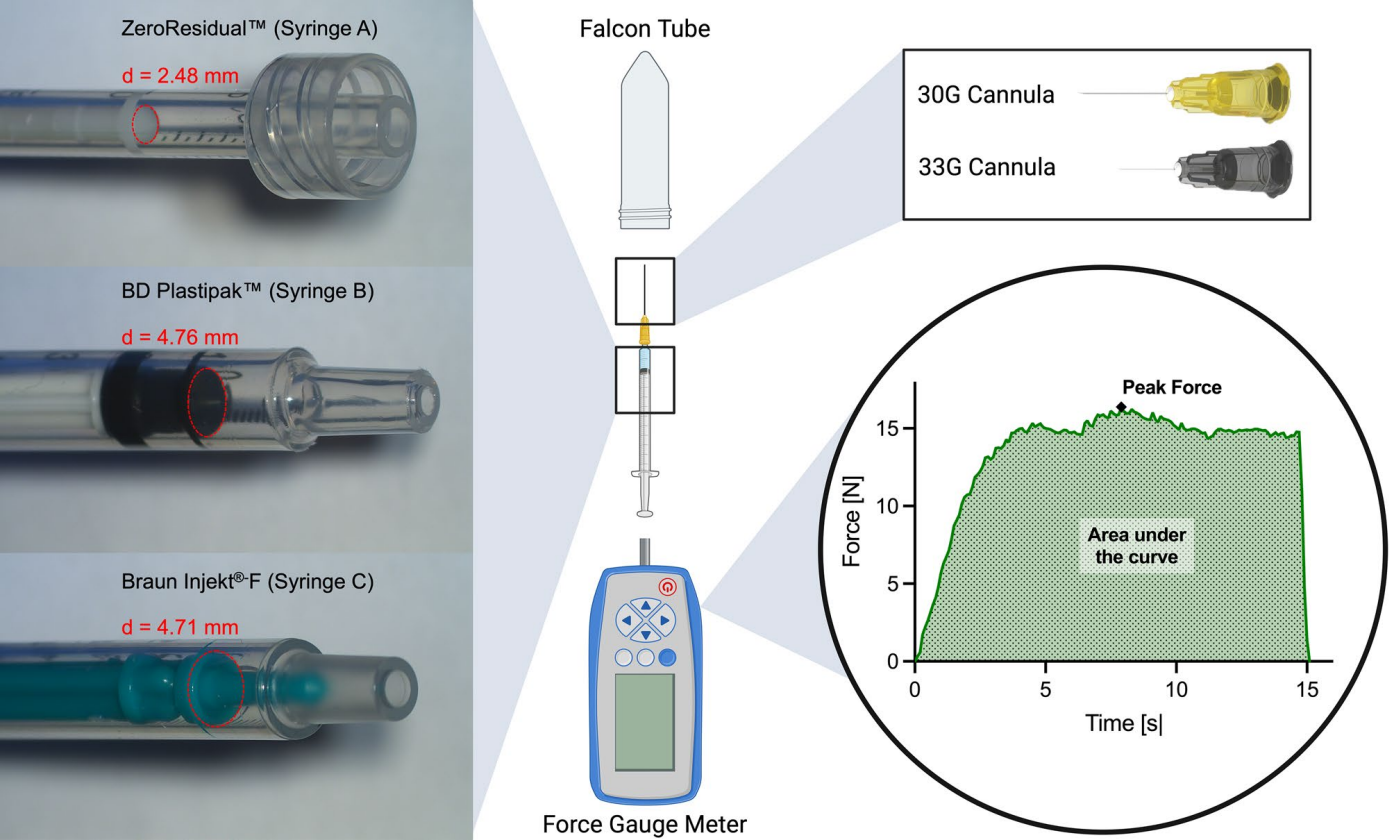
Bird’s eye perspective of the measurement procedure. Syringes were fixed in place while the syringe stamp was pushed with the force gauge meter. A Falcon tube served to catch the expelled liquid. The measured force was plotted against time to determine area under the curve and peak force. On the left, the three test syringes are displayed with their plungers at the 0.1 ml position and their diameters (d) highlighted in red

Figure 1 illustrates the injection procedures and the three different syringes with their respective plungers. Each syringe was stabilized by the examiner’s hand, and the force gauge meter was used to push the plunger until the entire fluid volume was expelled into a Falcon tube. A single examiner performed all five measurements in one session to minimize variability between injections.

### Statistical analyses

The AUC and peak force of each syringe were compared across both cannula sizes using a two-way ANOVA, followed by Tukey’s post-hoc test. *p*-values < 0.05 were considered statistically significant. Statistical analyses were performed using Prism 10.4.1 (GraphPad Prism. San Diego, CA, United States).

## Results

### Area under the curve

**Fig. 2:**
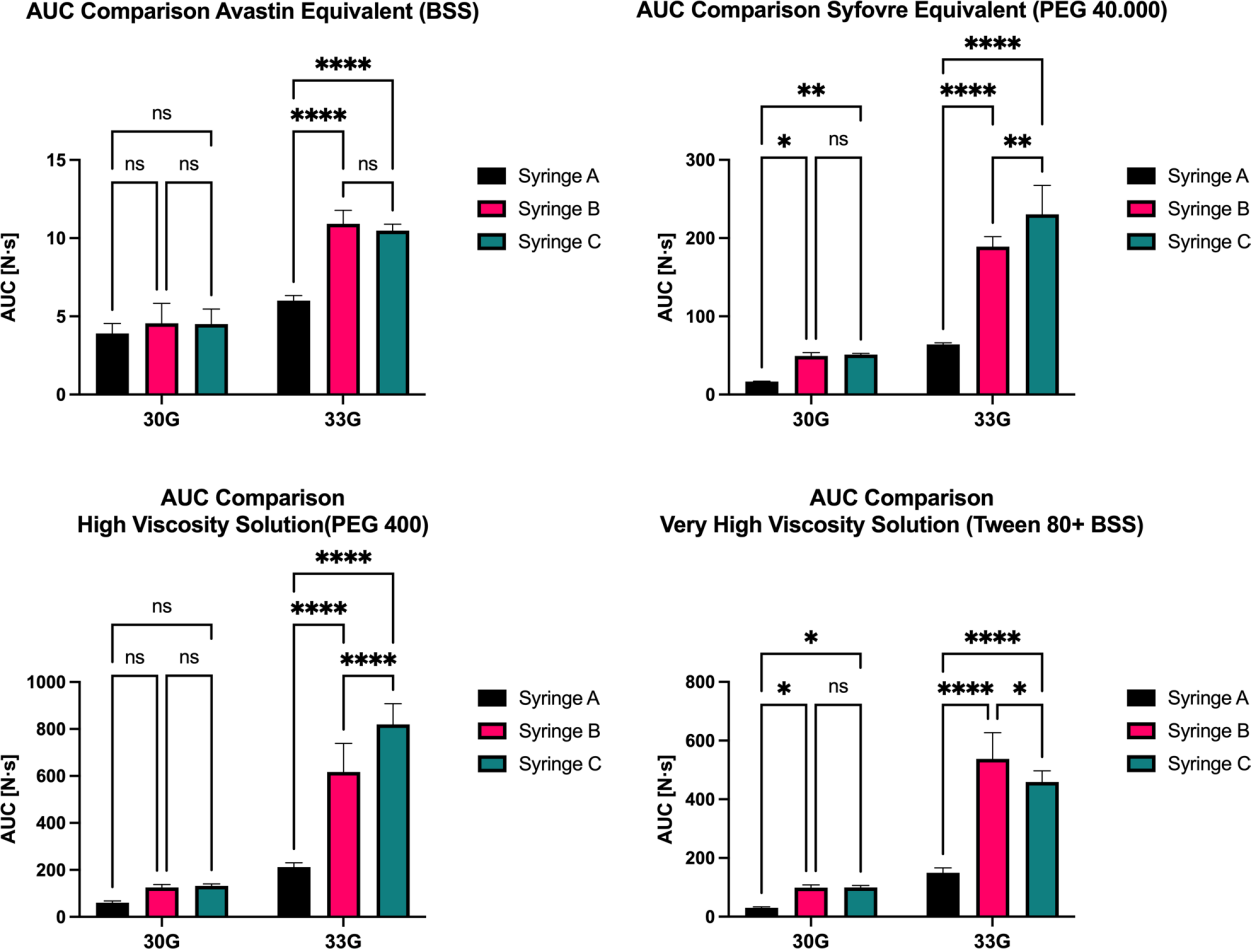
Comparison of the average area under the curve (AUC) of each combination. Whiskers on the bars represent the standard deviation. Results of the two-way ANOVA are displayed on the graphs. Significance markers: ns = not significant, *=*p*<0.05, **=*p*<0.01, ***=*p*<0.001, ****=*p*<0.0001. Abbreviations: AUC … area under the curve; BSS … Balanced Salt Solution; PEG … polyethylene glycol

Results are listed in Table 3 and illustrated in in Fig. 2. Descriptive statistics and results of the two-way ANOVA and Tukey’s post-hoc test are summarized in Tables S1, S2, and S3, respectively.

**Table 3:** Total area under the curve values

Cannula	Fluid	Syringe	1	2	3	4	5	⊘
30 G	BSS	Syringe C	5.026	5.983	4.131	4.002	3.457	4.520
		Syringe B	5.320	5.757	5.345	3.103	3.230	4.551
		Syringe A	4.996	3.803	3.687	3.585	3.388	3.892
	Diluted Tween	Syringe C	94.060	109.400	103.200	98.510	93.300	99.694
		Syringe B	84.440	105.700	102.200	93.760	108.300	98.880
		Syringe A	27.570	32.580	32.720	26.930	32.930	30.546
	PEG 400	Syringe C	121.100	142.800	129.900	137.700	128.900	132.080
		Syringe B	117.000	137.200	123.000	112.300	138.700	125.640
		Syringe A	49.930	58.000	61.200	67.350	66.520	60.600
	PEG 40.000	Syringe C	49.950	49.640	50.900	51.730	53.480	51.140
		Syringe B	46.960	55.460	49.910	50.510	43.520	49.272
		Syringe A	16.830	17.350	16.060	15.700	16.800	16.548
33 G	BSS	Syringe C	10.470	10.710	10.580	9.774	10.850	10.477
		Syringe B	11.180	11.950	10.260	9.806	11.390	10.917
		Syringe A	6.029	5.683	5.732	5.995	6.503	5.988
	Diluted Tween	Syringe C	470.600	398.300	501.700	463.600	462.100	459.260
		Syringe B	608.700	626.100	565.100	462.800	426.700	537.880
		Syringe A	144.300	162.900	171.400	136.700	131.800	149.420
	PEG 400	Syringe C	669.000	841.400	843.700	836.200	905.800	819.220
		Syringe B	444.400	536.000	683.600	728.200	694.900	617.420
		Syringe A	190.700	210.200	216.600	201.700	240.400	211.920
	PEG 40.000	Syringe C	271.000	268.000	188.800	216.600	207.000	230.280
		Syringe B	177.800	211.000	182.900	188.000	185.800	189.100
		Syringe A	66.080	62.520	66.320	62.920	63.030	64.174

All values are given in Newton-seconds [N.s]

Although not consistently statistically significant, Syringe A exhibited numerically lower AUC than the other two syringes. The difference was particularly pronounced with the smaller 33 G cannula, where Syringe A showed highly significant reductions in AUC across all fluids compared with Syringes B and C. With the 30 G cannula, a significant reduction was only observed for diluted Tween and PEG 40,000, the two most viscous solutions. While Syringes B and C performed similarly with no significant difference using 30 G cannulas, discrepancies emerged with the 33 G cannula; for diluted Tween, Syringe B exhibited a significantly higher AUC than Syringe C, whereas the opposite was true for PEG 400 and PEG 40,000. Injection of BSS showed no significant differences between Syringes B and C at either cannula size. Overall, injection resistance was consistently lowest for Syringe A and increased with smaller cannulas or higher viscosity solutions.

### Peak force

**Fig. 3:**
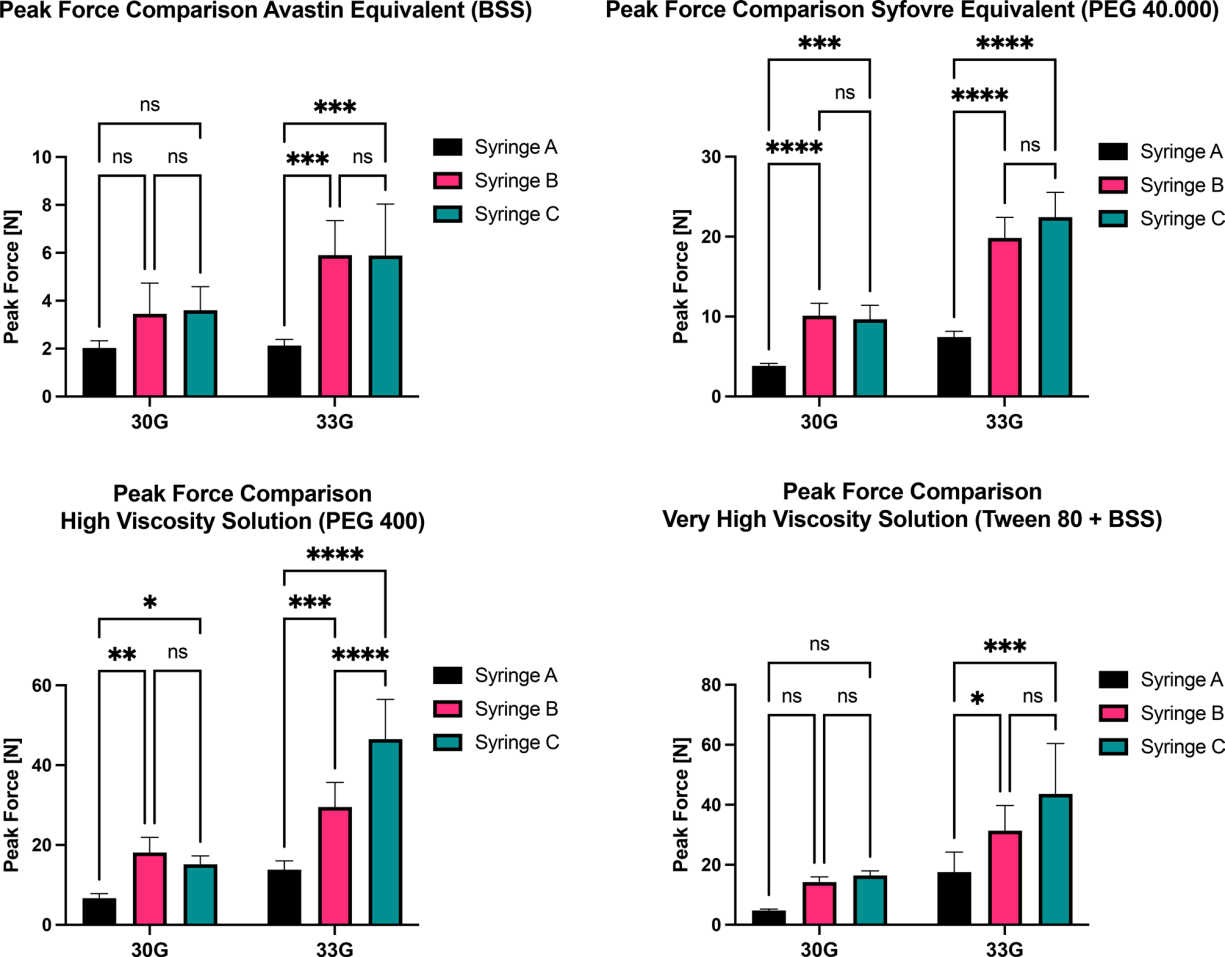
Comparison of the average peak force of each combination. Whiskers on the bars represent the standard deviation. Results of the two-way ANOVA are displayed on the graphs. Significance markers: ns = not significant, *=*p*<0.05, **=*p*<0.01, ***=*p*<0.001, ****=*p*<0.0001. Abbreviations: AUC … area under the curve; BSS … Balanced Salt Solution; PEG … polyethylene glycol

The maximum resistance force [N] during injection and the mean values for each group are presented in Table 4 and illustrated in Fig. 3. Descriptive statistics, along with the results of the two-way ANOVA and Tukey’s post-hoc test, are summarized in Tables S1, S2 and S3, respectively.

**Table 4:** Peak force measurements values

Cannula	Fluid	Syringe	1	2	3	4	5	⊘
30 G	BSS	Syringe C	4.180	4.730	3.940	2.380	2.790	3.604
		Syringe B	4.210	5.080	3.640	2.010	2.350	3.458
		Syringe A	2.380	1.830	1.870	2.330	1.740	2.030
	PEG 40.000	Syringe C	11.340	7.520	11.590	8.600	9.230	9.656
		Syringe B	9.160	11.740	9.830	11.620	8.180	10.106
		Syringe A	4.020	3.660	4.280	3.680	3.570	3.842
	PEG 400	Syringe C	14.480	12.620	15.160	15.400	18.340	15.200
		Syringe B	15.730	23.810	13.870	18.190	19.150	18.150
		Syringe A	5.500	5.770	6.360	8.220	7.540	6.678
	Diluted Tween	Syringe C	16.690	17.520	18.180	14.460	15.340	16.438
		Syringe B	14.510	16.920	14.330	12.490	12.830	14.216
		Syringe A	5.020	5.420	4.760	4.330	4.320	4.770
33 G	BSS	Syringe C	6.460	7.300	8.370	3.620	3.690	5.888
		Syringe B	7.570	6.010	6.970	4.050	4.900	5.900
		Syringe A	1.900	2.190	2.470	2.230	1.840	2.126
	PEG 40.000	Syringe C	25.560	25.930	19.620	21.380	19.660	22.430
		Syringe B	17.530	24.110	18.490	18.890	20.190	19.842
		Syringe A	7.030	6.820	8.570	7.730	6.980	7.426
	PEG 400	Syringe C	31.590	42.400	55.440	55.040	48.270	46.548
		Syringe B	30.150	39.740	26.680	23.870	27.180	29.524
		Syringe A	12.630	11.110	13.250	16.390	15.760	13.828
	Diluted Tween	Syringe C	52.950	46.800	64.970	26.100	27.370	43.638
		Syringe B	41.400	30.690	38.280	23.250	23.140	31.352
		Syringe A	21.520	20.660	24.580	11.760	9.260	17.556

All values are given in Newton [N]

Comparison of peak forces yielded similar results to those observed for the AUC. Syringe A generally exhibited lower average peak forces across all measurements, although differences were not statistically significant for BSS and diluted Tween injections with a 30 G cannula. Differences between Syringes B and C remained non-significant for injections with both cannula diameters, except for PEG 400 with a 33 G cannula, where Syringe B demonstrated a significantly lower peak force compared with Syringe C.

### Resistance force

**Fig. 4:**
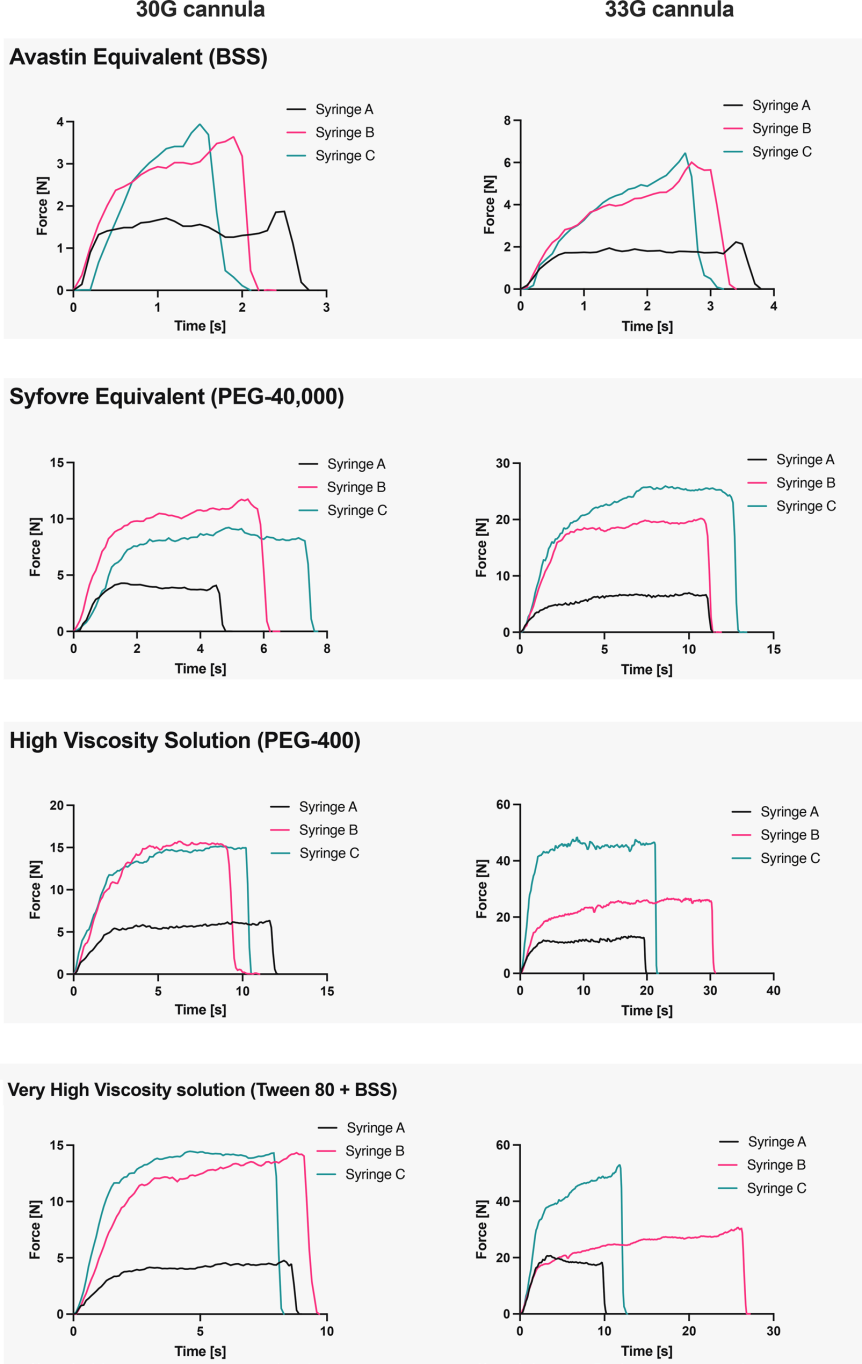
Force resistance measurements. For each combination of fluid and cannula, all three syringes force profiles are plotted on one graph. Abbreviations: BSS … Balanced Salt Solution; PEG … polyethylene glycol

Figure 4 illustrates the resistance force measurements over time for injections performed with the different syringes, fluids, and cannulas. As the injections were performed manually, the force profile varied between executions. From the five measurements, one representative curve was chosen.

Regardless of the injected fluid, all syringes exhibited a force-time profile characterized by a rapid rise in force, a plateau, and a quick decline towards zero at the end of the injection. Smaller cannula diameters led to longer injection times and higher peak forces, resulting in increased AUC. Although total injection time varied between syringes, no consistent pattern related to viscosity or cannula size was observed. Peak force and plateau were lower in all injections performed with Syringe A.

## Discussion

This study aimed to compare injection performance across three syringe models, measuring resistance force as the viscosity of the injected fluid was varied and using two cannula sizes.

We observed considerable differences between the two cannula sizes and among the three syringes in terms of peak force and AUC. Across all comparisons, Syringe A consistently exhibited lower peak forces and AUCs than the other two syringes, likely because of its smaller plunger diameter. Compared with Syringe C, Syringe B showed a significant reduction in AUC and peak force for certain liquids when used with the 33 G cannula. Differences in force were most pronounced with higher-viscosity liquids and the smaller 33 G cannula. As predicted from Hagen-Poiseuille’s law, decreasing the cannula diameter and increasing liquid viscosity increased the resistance force, although no clear correlation between viscosity and AUC or peak force was observed. These findings suggest that while silicone oil lubrication influences injection force, syringe-plunger diameter has a far stronger impact than lubrication alone. Though lubrication reduces the friction at the plunger-syringe interface, this effect appears to only deliver marginal returns, specifically the plunger area causes disproportionate changes that ultimately outweigh smaller effects of lubrication.

Furthermore, lubrication with silicone oil carries its own risks, as it has been shown to leach into drug formulations and has been found in the vitreous patients following injection. [10, 11] In-vitro studies demonstrate that silicone oil droplets can induce protein aggregation [12–14], potentially causing complications such as floaters. Moreover, silicone oil-coated syringes have been associated with higher rates of post-injection inflammatory reactions [15], leading to the recommendation that these syringes should not be used for IVI [16].

In light of these concerns as well as the results of this study, plunger diameter seems to not only be the more effective, but also the safer option to reduce injection force for intravitreal drug delivery.

Some limitations to our study should be considered. Although the measurements were performed in a single session and attempts were made to maintain consistent injection speed, total injection times and peak forces varied, even within repeated measurements for the same combination of fluid and cannula. We chose the manual approach to better reflect clinical practice; however, to reduce such variability, a mechanized setup could be used, allowing for strict control of injection time. Under the circumstances of this study, area under the force-time curve was analyzed to mitigate this variation. Since the applied force over the injection period was integrated, longer injections with less force and shorter injections with higher force yielded comparable results. An example of this is illustrated in Fig. S1.

Additionally, measurements may differ when injecting into a patient’s eye rather than merely expelling liquid from the syringe. Given the manual setup and the high number of measurements, this approach was not a feasible one in the current study; for instance, future research could explore this with a smaller sample size of porcine cadaver eyes. Previous work on IOL injectors showed no difference between porcine eye and a plastic dish control in most injection systems [9], though this should be assessed for intravitreal injections as well.

It is important to recognize that our data serve as a starting point for future research and cannot be directly translated into clinical practice. Injection force only represents one part of the operational experience, and if an optimal syringe is to be determined, other factors such as injection comfort have to be acknowledged. Another important issue, specifically relevant with high drug concentrations, high viscosity and small volumes, is the post-injection drug residue in the syringes. Previous studies compared residual volumes across multiple syringes used for IVI, including the ones assessed in this study [17, 18] with Syringe A showing the lowest residual volumes. Expanding on that work with a broader range of fluids of different viscosities could add to the current literature and aid with the applicability of our data in a real-world setting.

While this study allowed to find significant differences between currently available syringes, transferring these findings to designing an “ideal” syringe for IVI would require analyzing further aspects of the design such as inner wall coating or plunger material.

In conclusion, of the three syringes we examined, the syringe with smaller plunger diameter, Syringe A, consistently showed the lowest peak force and AUC, with differences being most pronounced for high-viscosity fluids combined with small cannulas. While a smaller plunger diameter reducing the needed injection force is an expected result on its own, our data also suggest that it is the main factor influencing injection force when needle gauge and drug viscosity are fixed. Even though silicone oil lubrication also slightly reduced injection force, the plunger diameter had a far greater effect. Given the previously mentioned issues arising from silicone oil lubrication, plunger diameter appears to be the safer and more effective way to reduce the applied force during injection. Using a syringe with smaller plunger diameter may therefore provide the most benefit when administrating viscous drugs through narrow-gauge cannulas.

## Electronic supplementary material

Below is the link to the electronic supplementary material.

Supplementary Material 1

Supplementary Material 2

Supplementary Material 3

## Data Availability

The data are available from the corresponding author upon reasonable request.
