# Supplementary figures and images for: The effect of syringe design and cannula dimensions on time-force curve in intravitreal injection across different drug viscosities: area under the curve and peak injection force
Source: Int J Retina Vitreous. 2026 Mar 20;12:57. doi: 10.1186/s40942-026-00833-2 (PMC13063457; doi:10.1186/s40942-026-00833-2)

### Force-Time Curve

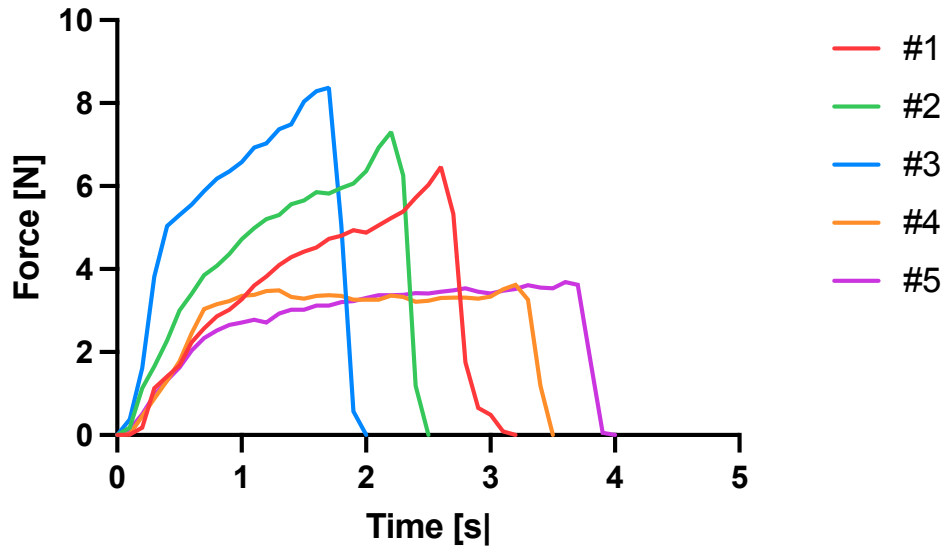

### AUC

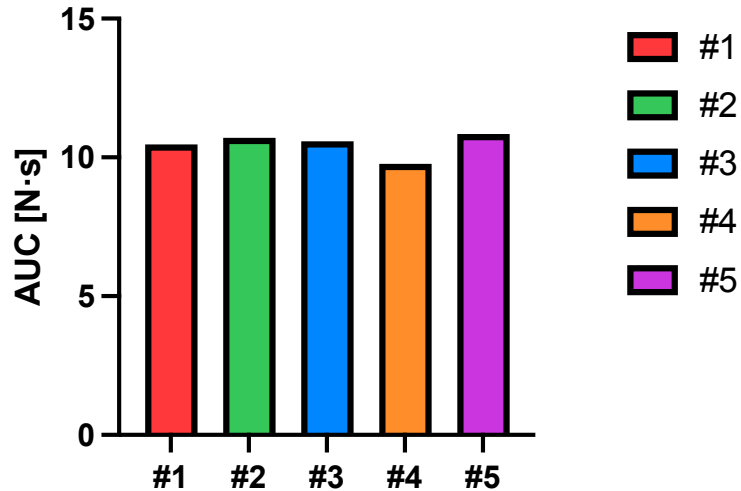

Supplement: Supplementary file 1 — Supplementary Material 1 [file 40942_2026_833_MOESM1_ESM.pdf]
