# Supplementary material for: The effect of syringe design and cannula dimensions on time-force curve in intravitreal injection across different drug viscosities: area under the curve and peak injection force
Source: Int J Retina Vitreous. 2026 Mar 20;12:57. doi: 10.1186/s40942-026-00833-2 (PMC13063457; doi:10.1186/s40942-026-00833-2)
Supplement: Supplementary file 2 — Supplementary Material 2 [file 40942_2026_833_MOESM2_ESM.docx]

**Figure S1.** AUCs of Different Force-Time Curves. Despite the repeated measurement of force over time showing sizable variation, their AUCs remain within a small range, illustrating how AUC can enable comparable results despite varying injection profiles. Shown measurements are from expelling BSS through a 33G cannula using Syringe C.
