# Supplementary material for: The effect of syringe design and cannula dimensions on time-force curve in intravitreal injection across different drug viscosities: area under the curve and peak injection force
Source: Int J Retina Vitreous. 2026 Mar 20;12:57. doi: 10.1186/s40942-026-00833-2 (PMC13063457; doi:10.1186/s40942-026-00833-2)
Supplement: Supplementary file 3 — Supplementary Material 3 [file 40942_2026_833_MOESM3_ESM.docx]

**Table S1.** Descriptive statistics of AUC recordings.

| **Cannula size** | **Syringe** | **Descriptive statistics** | **Liquids** | | | |
| --- | --- | --- | --- | --- | --- | --- |
|  |  |  | **BSS** | **PEG 40,000** | **PEG 400** | **Tween** |
| 30G | Syringe A | Number of values | 5 | 5 | 5 | 5 |
|  |  | Mean | 3.892 | 16.548 | 60.600 | 30.546 |
|  |  | Std. Deviation | 0.636 | 0.660 | 7.099 | 3.020 |
|  |  | Std. Error of Mean | 0.284 | 0.295 | 3.175 | 1.351 |
|  |  | 95% CI | 3.102 to 4.681 | 15.728 to 17.368 | 51.786 to 69.414 | 26.796 to 34.296 |
|  | Syringe B | Number of values | 5 | 5 | 5 | 5 |
|  |  | Mean | 4.551 | 49.272 | 125.640 | 98.880 |
|  |  | Std. Deviation | 1.277 | 4.435 | 11.872 | 9.760 |
|  |  | Std. Error of Mean | 0.571 | 1.983 | 5.409 | 4.365 |
|  |  | 95% CI | 2.966 to 6.136 | 43.766 to 54.778 | 110.899 to 140.381 | 86.762 to 110.998 |
|  | Syringe C | Number of values | 5 | 5 | 5 | 5 |
|  |  | Mean | 4.504 | 51.140 | 132.080 | 99.694 |
|  |  | Std. Deviation | 0.959 | 1.545 | 8.395 | 6.718 |
|  |  | Std. Error of Mean | 0.429 | 0.691 | 3.755 | 3.004 |
|  |  | 95% CI | 3.313 to 5.695 | 49.222 to 53.058 | 121.656 to 142.504 | 91.353 to 108.035 |
| 33G | Syringe A | Number of values | 5 | 5 | 5 | 5 |
|  |  | Mean | 5.988 | 64.174 | 211.920 | 149.420 |
|  |  | Std. Deviation | 0.326 | 1.861 | 18.646 | 17.054 |
|  |  | Std. Error of Mean | 0.146 | 0.832 | 8.339 | 7.627 |
|  |  | 95% CI | 5.583 to 6.393 | 61.863 to 66.485 | 188.768 to 235.072 | 128.245 to 170.595 |
|  | Syringe B | Number of values | 5 | 5 | 5 | 5 |
|  |  | Mean | 10.917 | 189.100 | 617.420 | 537.880 |
|  |  | Std. Deviation | 0.870 | 12.824 | 121.676 | 88.794 |
|  |  | Std. Error of Mean | 0.389 | 5.735 | 54.415 | 39.710 |
|  |  | 95% CI | 9.837 to 1.997 | 173.177 to 205.023 | 466.339 to 768.501 | 427.628 to 648.132 |
|  | Syringe C | Number of values | 5 | 5 | 5 | 5 |
|  |  | Mean | 10.477 | 230.280 | 819.220 | 459.260 |
|  |  | Std. Deviation | 0.418 | 37.184 | 88.659 | 37.659 |
|  |  | Std. Error of Mean | 0.187 | 16.629 | 39.650 | 16.842 |
|  |  | 95% CI | 9.958 to 10.996 | 184.110 to 276.450 | 709.135 to 929.305 | 412.500 to 506.020 |

Mean, SD, SEM and 95% CI given in [N·s].

**Table S2.** Results of the two-way ANOVA performed, analyzing effects of cannulas, syringes and their interaction on area under the curve of the injection.

| **Effect** | | **F (Dfn, DFd)** | **p-value** | **Partial η²** |
| --- | --- | --- | --- | --- |
| BSS | Interaction | F (2, 24) = 20.93 | p<0.0001 | 0.64 |
|  | Cannula | F (1, 24) = 261.00 | p<0.0001 | 0.92 |
|  | Syringe | F (2, 24) = 36.00 | p<0.0001 | 0.75 |
| PEG 40,000 | Interaction | F (2, 24) = 43.45 | p<0.0001 | 0.78 |
|  | Cannula | F (1, 24) = 427.20 | p<0.0001 | 0.95 |
|  | Syringe | F (2, 24) = 106.50 | p<0.0001 | 0.90 |
| PEG 400 | Interaction | F (2, 24) = 47.39 | p<0.0001 | 0.80 |
|  | Cannula | F (1, 24) = 380.10 | p<0.0001 | 0.94 |
|  | Syringe | F (2, 24) = 77.93 | p<0.0001 | 0.87 |
| Tween + BSS | Interaction | F (2, 24) = 42.78 | p<0.0001 | 0.78 |
|  | Cannula | F (1, 24) = 432.00 | p<0.0001 | 0.95 |
|  | Syringe | F (2, 24) = 91.95 | p<0.0001 | 0.88 |

**Table S3.** Results of Tukey’s post-hoc test of area under the curve.

| **Comparison** | | | **Mean difference** | **95% CI of diff.** | **Adjusted p-Value** |
| --- | --- | --- | --- | --- | --- |
| BSS | 30G | Syringe A vs. Syringe B | -0.6592 | -1.947 to 0.6290 | 0.4209 |
|  |  | Syringe A vs. Syringe C | -0.6120 | -1.900 to 0.6762 | 0.4725 |
|  |  | Syringe B vs. Syringe C | 0.04720 | -1.241 to 1.335 | 0.9954 |
|  | 33G | Syringe A vs. Syringe B | -4.929 | -6.217 to  -3.641 | <0.0001 |
|  |  | Syringe A vs. Syringe C | -4.488 | -5.777 to  -3.200 | <0.0001 |
|  |  | Syringe B vs. Syringe C | 0.4404 | -0.8478 to 1.729 | 0.6739 |
| PEG 40,000 | 30G | Syringe A vs. Syringe B | -32.72 | -58.30 to  -7.150 | 0.0105 |
|  |  | Syringe A vs. Syringe C | -34.59 | -60.17 to  -9.018 | 0.0068 |
|  |  | Syringe B vs. Syringe C | -1.868 | -27.44 to 23.71 | 0.9818 |
|  | 33G | Syringe A vs. Syringe B | -124.9 | -150.5 to  -99.35 | <0.0001 |
|  |  | Syringe A vs. Syringe C | -166.1 | -191.7 to  -140.5 | <0.0001 |
|  |  | Syringe B vs. Syringe C | -41.18 | -66.75 to  -15.61 | 0.0014 |
| PEG 400 | 30G | Syringe A vs. Syringe B | -65.04 | -163.4 to 33.33 | 0.2444 |
|  |  | Syringe A vs. Syringe C | -71.48 | -169.9 to 26.89 | 0.1863 |
|  |  | Syringe B vs. Syringe C | -6.440 | -104.8 to 91.93 | 0.9854 |
|  | 33G | Syringe A vs. Syringe B | -405.5 | -503.9 to  -307.1 | <0.0001 |
|  |  | Syringe A vs. Syringe C | -607.3 | -705.7 to  -508.9 | <0.0001 |
|  |  | Syringe B vs. Syringe C | -201.8 | -300.2 to  -103.4 | <0.0001 |
| Tween + BSS | 30G | Syringe A vs. Syringe B | -68.33 | -132.0 to  -4.689 | 0.0337 |
|  |  | Syringe A vs. Syringe C | -69.15 | -132.8 to  -5.503 | 0.0314 |
|  |  | Syringe B vs. Syringe C | -0.8140 | -64.46 to 62.83 | 0.9994 |
|  | 33G | Syringe A vs. Syringe B | -388.5 | -452.1 to  -324.8 | <0.0001 |
|  |  | Syringe A vs. Syringe C | -309.8 | -373.5 to  -246.2 | <0.0001 |
|  |  | Syringe B vs. Syringe C | 78.62 | 14.97 to 142.3 | 0.0135 |

Mean difference and 95% CI given in [N·s].

**Table S4.** Descriptive statistics of Peak Force Measurements.

| **Cannula size** | **Syringe** | **Descriptive statistics** | **Liquids** | | | |
| --- | --- | --- | --- | --- | --- | --- |
|  |  |  | **BSS** | **PEG 40,000** | **PEG 400** | **Tween** |
| 30G | Syringe A | Number of values | 5 | 5 | 5 | 5 |
|  |  | Mean | 2.030 | 3.842 | 6.678 | 4.770 |
|  |  | Std. Deviation | 0.301 | 0.299 | 1.166 | 0.469 |
|  |  | Std. Error of Mean | 0.135 | 0.134 | 0.521 | 0.210 |
|  |  | 95% CI | 1.656 to 2.404 | 3.471 to 4.213 | 5.231 to 8.125 | 4.187 to 5.353 |
|  | Syringe B | Number of values | 5 | 5 | 5 | 5 |
|  |  | Mean | 3.458 | 10.106 | 18.150 | 14.216 |
|  |  | Std. Deviation | 1.280 | 1.553 | 3.782 | 1.754 |
|  |  | Std. Error of Mean | 0.572 | 0.694 | 1.691 | 0.785 |
|  |  | 95% CI | 1.869 to 5.047 | 8.178 to 12.034 | 13.454 to 22.846 | 12.038 to 16.394 |
|  | Syringe C | Number of values | 5 | 5 | 5 | 5 |
|  |  | Mean | 3.604 | 9.656 | 15.200 | 16.438 |
|  |  | Std. Deviation | 0.984 | 1.763 | 2.066 | 1.532 |
|  |  | Std. Error of Mean | 0.440 | 0.789 | 0.924 | 0.685 |
|  |  | 95% CI | 2.382 to 4.826 | 7.467 to 11.845 | 12.635 to 17.765 | 14.536 to 18.340 |
| 33G | Syringe A | Number of values | 5 | 5 | 5 | 5 |
|  |  | Mean | 2.126 | 7.426 | 13.828 | 17.556 |
|  |  | Std. Deviation | 0.258 | 0.729 | 2.205 | 6.654 |
|  |  | Std. Error of Mean | 0.115 | 0.326 | 0.986 | 2.976 |
|  |  | 95% CI | 1.806 to 2.446 | 6.521 to 8.331 | 11.090 to 16.566 | 9.294 to 25.818 |
|  | Syringe B | Number of values | 5 | 5 | 5 | 5 |
|  |  | Mean | 5.900 | 19.842 | 29.524 | 31.352 |
|  |  | Std. Deviation | 1.446 | 2.570 | 6.130 | 8.404 |
|  |  | Std. Error of Mean | 0.647 | 1.149 | 2.741 | 3.758 |
|  |  | 95% CI | 4.104 to 7.696 | 16.651 to 23.033 | 21.913 to 37.135 | 20.918 to 41.786 |
|  | Syringe C | Number of values | 5 | 5 | 5 | 5 |
|  |  | Mean | 5.888 | 22.430 | 46.548 | 43.638 |
|  |  | Std. Deviation | 2.148 | 3.111 | 9.938 | 16.763 |
|  |  | Std. Error of Mean | 0.961 | 1.391 | 4.445 | 7.497 |
|  |  | 95% CI | 3.221 to 8.555 | 18.567 to 26.293 | 34.208 to 58.888 | 22.824 to 64.452 |

Mean, SD, SEM and 95%CI in [N].

**Table S5.** Results of the two-way ANOVA performed, analyzing effects of cannulas, syringes and their interaction on peak force during injection.

| **Effect** | | **F (Dfn, DFd)** | **p-value** | **Partial η²** |
| --- | --- | --- | --- | --- |
| BSS | Interaction | F (2, 24) = 2.723 | p=0.0859 | 0.18 |
|  | Cannula | F (1, 24) = 12.28 | p=0.0018 | 0.34 |
|  | Syringe | F (2, 24) = 14.67 | p<0.0001 | 0.55 |
| PEG 40,000 | Interaction | F (2, 24) = 14.66 | p<0.0001 | 0.55 |
|  | Cannula | F (1, 24) = 151.8 | p<0.0001 | 0.86 |
|  | Syringe | F (2, 24) = 87.73 | p<0.0001 | 0.88 |
| PEG 400 | Interaction | F (2, 24) = 15.55 | p<0.0001 | 0.56 |
|  | Cannula | F (1, 24) = 77.17 | p<0.0001 | 0.76 |
|  | Syringe | F (2, 24) = 40.91 | p<0.0001 | 0.77 |
| Tween + BSS | Interaction | F (2, 24) = 2.042 | p=0.1517 | 0.15 |
|  | Cannula | F (1, 24) = 40.63 | p<0.0001 | 0.63 |
|  | Syringe | F (2, 24) = 13.55 | p=0.0001 | 0.53 |

**Table S6.** Results of Tukey’s post-hoc test on peak force during injection.

| **Comparison** | | | **Mean difference** | **95% CI of diff.** | **Adjusted p-Value** |
| --- | --- | --- | --- | --- | --- |
| BSS | 30G | Syringe A vs. Syringe B | -1.428 | -3.412 to 0.5563 | 0.1919 |
|  |  | Syringe A vs. Syringe C | -1.574 | -3.558 to 0.4103 | 0.1386 |
|  |  | Syringe B vs. Syringe C | -0.146 | -2.130 to 1.838 | 0.9816 |
|  | 33G | Syringe A vs. Syringe B | -3.774 | -5.758 to  -1.790 | 0.0002 |
|  |  | Syringe A vs. Syringe C | -3.762 | -5.746 to  -1.778 | 0.0002 |
|  |  | Syringe B vs. Syringe C | 0.012 | -1.972 to 1.996 | 0.9999 |
| PEG 40,000 | 30G | Syringe A vs. Syringe B | -6.264 | -9.317 to  -3.211 | <0.0001 |
|  |  | Syringe A vs. Syringe C | -5.814 | -8.867 to  -2.761 | 0.0002 |
|  |  | Syringe B vs. Syringe C | 0.4500 | -2.603 to 3.503 | 0.9283 |
|  | 33G | Syringe A vs. Syringe B | -12.42 | -15.47 to  -9.363 | <0.0001 |
|  |  | Syringe A vs. Syringe C | -15.00 | -18.06 to  -11.95 | <0.0001 |
|  |  | Syringe B vs. Syringe C | -2.588 | -5.641 to 0.4653 | 0.1076 |
| PEG 400 | 30G | Syringe A vs. Syringe B | -11.47 | -19.66 to  -3.287 | 0.0050 |
|  |  | Syringe A vs. Syringe C | -8.522 | -16.71 to  -0.3368 | 0.0402 |
|  |  | Syringe B vs. Syringe C | 2.950 | -5.235 to 11.14 | 0.6455 |
|  | 33G | Syringe A vs. Syringe B | -15.70 | -23.88 to  -7.511 | 0.0002 |
|  |  | Syringe A vs. Syringe C | -32.72 | -40.91 to  -24.53 | <0.0001 |
|  |  | Syringe B vs. Syringe C | -17.02 | -25.21 to  -8.839 | <0.0001 |
| Tween + BSS | 30G | Syringe A vs. Syringe B | -9.446 | -22.37 to 3.475 | 0.1827 |
|  |  | Syringe A vs. Syringe C | -11.67 | -24.59 to 1.253 | 0.0821 |
|  |  | Syringe B vs. Syringe C | -2.222 | -15.14 to 10.70 | 0.9037 |
|  | 33G | Syringe A vs. Syringe B | -13.80 | -26.72 to  -0.8753 | 0.0348 |
|  |  | Syringe A vs. Syringe C | -26.08 | -39.00 to  -13.16 | 0.0001 |
|  |  | Syringe B vs. Syringe C | -12.29 | -25.21 to 0.6347 | 0.0645 |

Mean difference and 95% CI in [N].
